# Supplementary material for: Integrated Bioinformatic Investigation of EXOSCs in Hepatocellular Carcinoma Followed by the Preliminary Validation of EXOSC5 in Cell Proliferation
Source: Int J Mol Sci. 2022 Oct 12;23(20):12161. doi: 10.3390/ijms232012161 (PMC9603681; doi:10.3390/ijms232012161)
Supplement: Supplementary file 1 [file ijms-23-12161-s001.zip › ijms-1911908-supplementary.pdf]

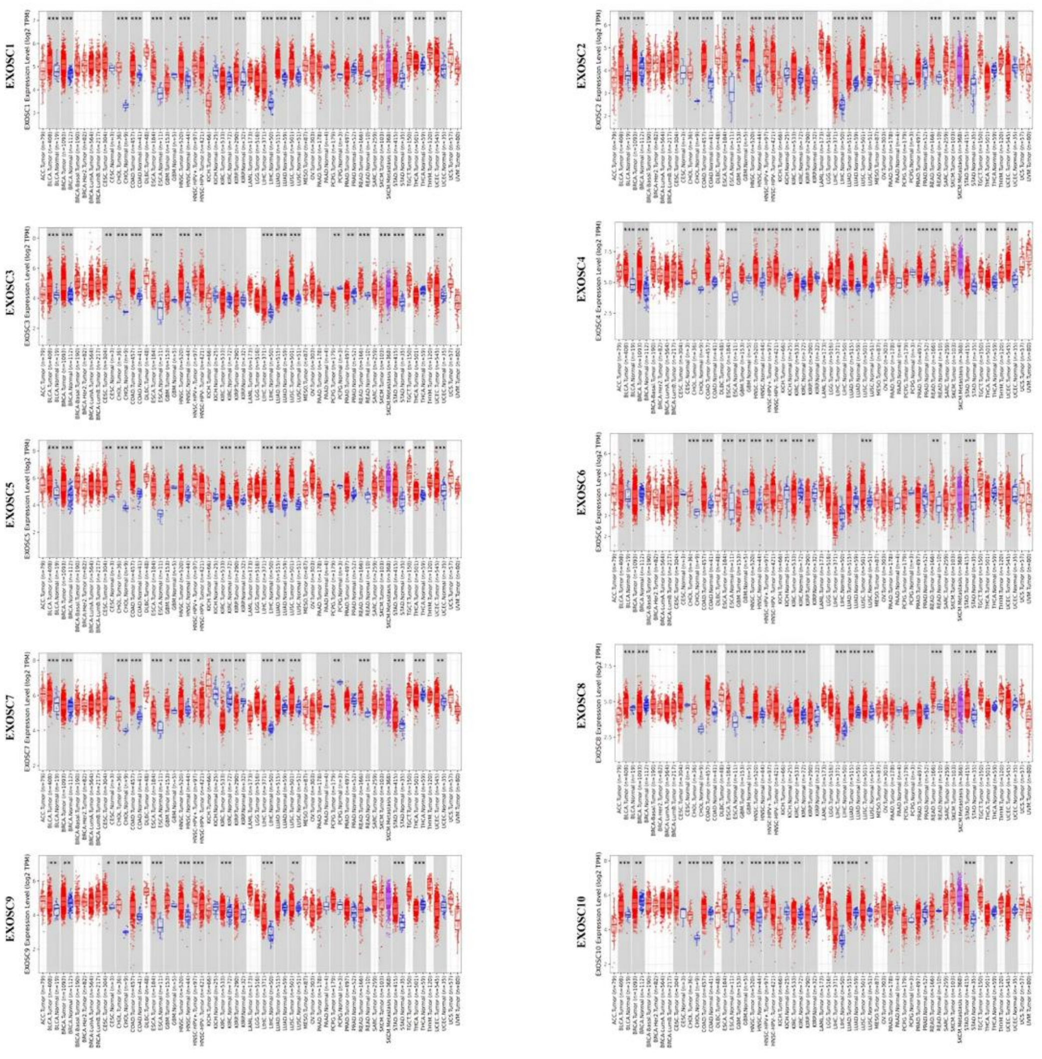

Figure S1. The expression level of the EXOSCs in different tumor tissues and para-cancerous tissues in the TCGA database were determined by TIMER.

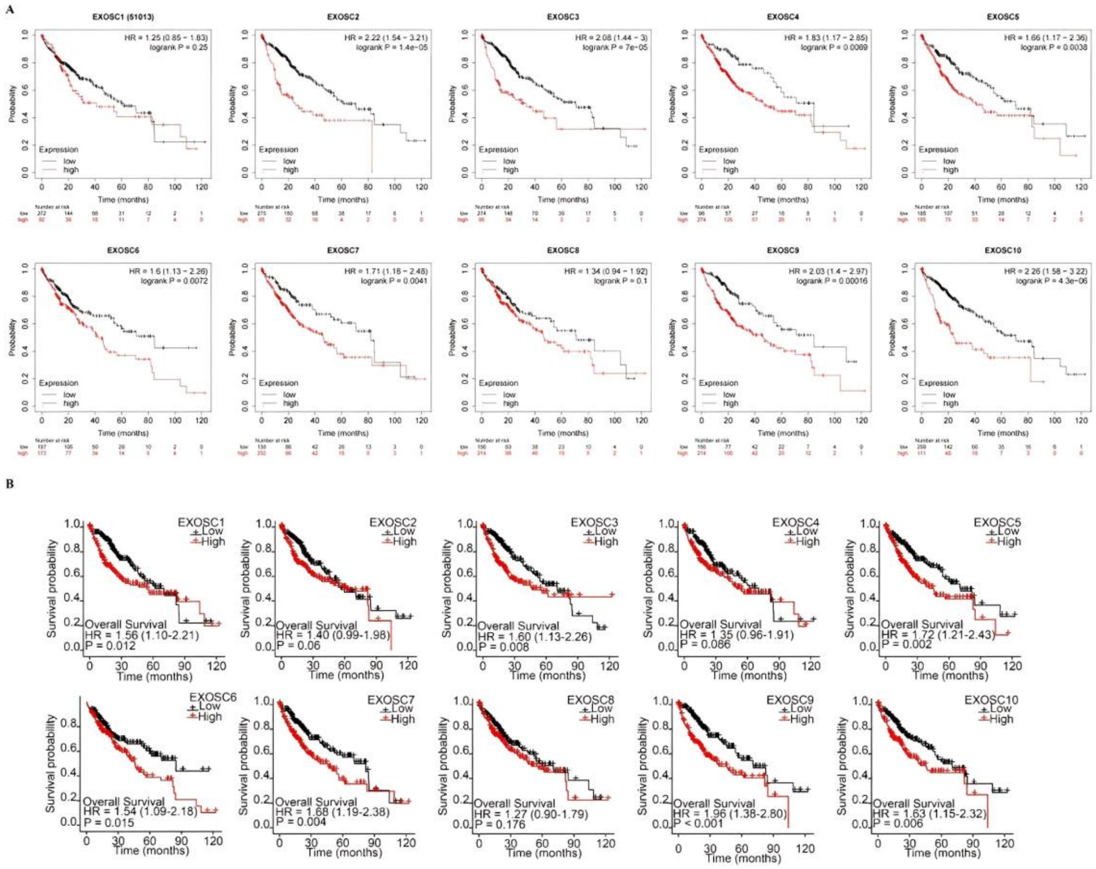

Figure S2. Prognostic characteristics of EXOSCs in patients with HCC.

(**A**) Up-regulated EXOSCs was associated with shorter OS, except EXOSC1/8 (TCGA database).

(**B**) Higher mRNA expressions of EXOSCs were significantly associated with poor OS, except EXOSC2/4/8 (Kaplan-Meier plotter).

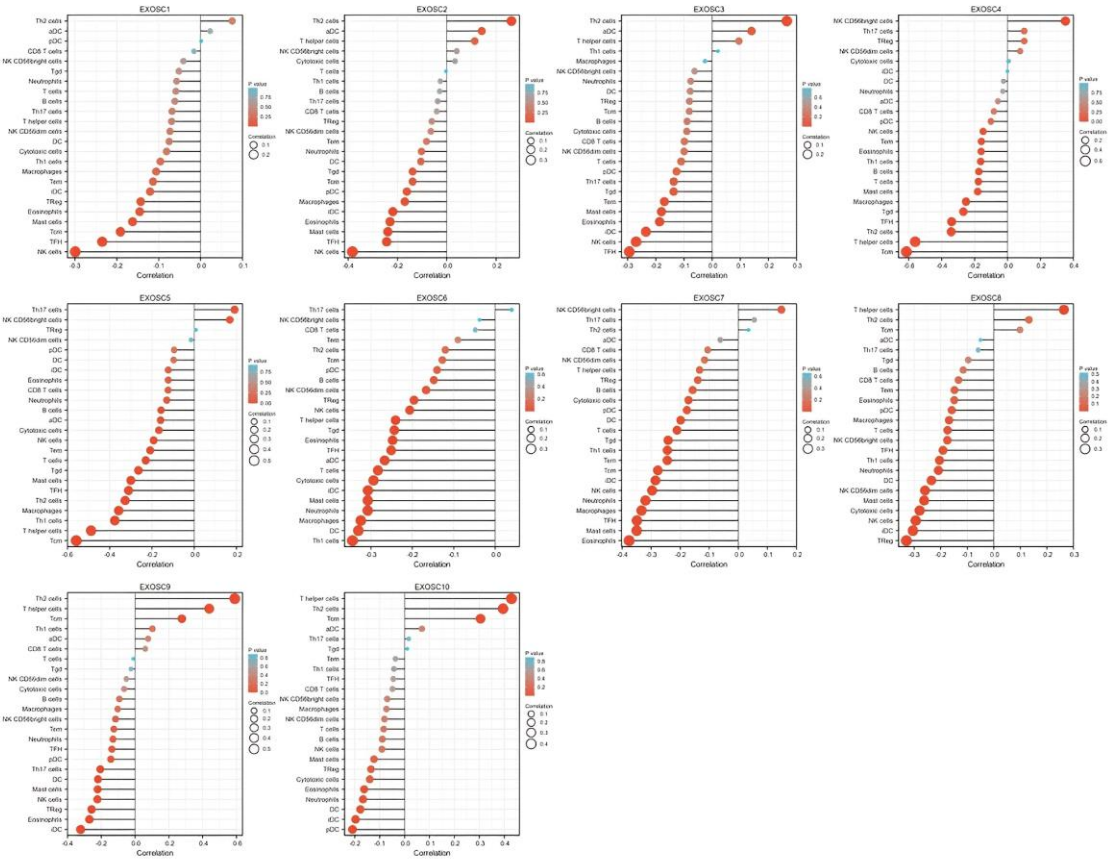

Figure S3. Analysis the correlation between EXOSCs expression and diverse immune cell infiltration.
